# From Sound to Meaning: Navigating Wernicke's Area in Language Processing

**DOI:** 10.7759/cureus.69833

**Published:** 2024-09-21

**Authors:** Pinaki D Wani

**Affiliations:** 1 Physiology, AIIMS Raebareli, Raebareli, IND

**Keywords:** language comprehension, language development, language disorders, neuroimaging, neuroplasticity, rehabilitation, wernicke's aphasia, wernicke's area

## Abstract

Wernicke's area, a critical brain region associated with language comprehension, was first identified by Carl Wernicke in the late 19th century. Situated in the left hemisphere’s posterior superior temporal gyrus, this area is essential for processing auditory and visual language inputs. It integrates semantic and syntactic information, playing a key role in meaningful communication.

The development of Wernicke's area during infancy and childhood is marked by rapid growth and refinement influenced by early language exposure and environmental stimuli. Neuroplasticity, the brain's ability to reorganize and adapt, is crucial for recovery from language impairments such as Wernicke's aphasia. This capacity for reorganization includes synaptic plasticity and axonal sprouting, which facilitate recovery through targeted rehabilitation and enriched environments. Recent research utilizing advanced neuroimaging and neuroanatomical tracing techniques has elucidated the connectivity of Wernicke's area with other language-related regions, such as Broca's area. Functional studies have revealed its specialized roles in processing different aspects of language, including phonological, semantic, and syntactic features. Moreover, investigations into language disorders and potential therapeutic interventions underscore the importance of harnessing neuroplasticity for effective treatment.

Emerging technologies, such as non-invasive brain stimulation and multimodal imaging, offer promising avenues for further exploration of Wernicke's area and its role in language functions. These innovations hold the potential to enhance our understanding of language processing and improve therapeutic strategies for language impairments.

In conclusion, Wernicke's area is central to language comprehension, and genetic and environmental factors influence its development. Understanding neuroplasticity and leveraging advanced research technologies can significantly advance our ability to address language-related disorders and enhance patient outcomes.

## Introduction and background

Wernicke's area is a crucial region of the brain associated with language comprehension. It was first identified and extensively studied by the German neurologist Carl Wernicke in the late 19th century. This area plays a fundamental role in the comprehension of spoken and written language. Wernicke's area is primarily located in the posterior part of the superior temporal gyrus, situated in the left hemisphere of the brain, specifically in the dominant hemisphere for language processing in most individuals [1]. It encompasses portions of the temporal and parietal lobes, with its boundaries somewhat variable among individuals but generally encompassing the posterior part of the superior temporal gyrus and adjacent areas [2]. Carl Wernicke first described this area in his influential 1874 publication, "The Symptom Complex of Aphasia: A Psychological Study on an Anatomical Basis". In this work, Wernicke observed patients with receptive aphasia, a condition characterized by difficulty in understanding language despite intact speech production. Wernicke's meticulous clinical observations led him to identify a distinct area in the left hemisphere associated with receptive language deficits. He noticed that lesions in specific regions of the left hemisphere resulted in profound impairments in language comprehension, while speech production remained relatively intact. His pioneering research laid the foundation for subsequent investigations into the neuroanatomical basis of language processing [3]. Wernicke's observations paved the way for further exploration into the functional and anatomical connectivity of brain regions involved in language processing. Subsequent neuroimaging studies, such as functional magnetic resonance imaging (fMRI) and positron emission tomography (PET), have provided additional insights into the intricate networks underlying language comprehension and production. These modern imaging techniques have corroborated Wernicke's initial observations and refined our understanding of the neural substrates of language.

## Review

Anatomy of Wernicke's area

Wernicke's area is primarily located in the left hemisphere of the brain, specifically in the posterior part of the superior temporal gyrus (STG). It extends into the supramarginal gyrus and angular gyrus of the parietal lobe. This region is predominantly found in the posterior part of the left superior temporal gyrus, though individual variations in its exact location do exist [4].

Microscopic structure

Microscopically, Wernicke's area consists of layers of neurons with distinct functional properties. These neurons are densely packed and exhibit complex interconnections, forming neural circuits that facilitate the processing of linguistic stimuli [5]. The area also contains specialized cell types, such as pyramidal cells, which are involved in integrating and transmitting information across different cortical regions.

Connections with other brain regions

Wernicke's area is interconnected with several brain regions involved in language processing, forming a complex network essential for the comprehension and production of speech:

Broca's Area

Broca's area, located in the posterior part of the inferior frontal gyrus, is responsible for speech production and articulation. It is closely connected to Wernicke's area via the arcuate fasciculus, a white matter tract that facilitates the transmission of information between the two regions. This connection enables the seamless integration of language comprehension and production processes [6].

Arcuate Fasciculus

The arcuate fasciculus is a bundle of nerve fibers that connects Wernicke's area with Broca's area. It serves as the primary pathway for the transmission of language-related information, facilitating the conversion of auditory input into articulated speech. Damage to the arcuate fasciculus can result in conduction aphasia, characterized by difficulties in repeating spoken language despite intact comprehension and production abilities [7].

Primary Auditory Cortex

The primary auditory cortex, located in the superior temporal gyrus, receives auditory input from the thalamus and is involved in the initial processing of sound stimuli. It forms reciprocal connections with Wernicke's area, allowing for the integration of auditory information with language comprehension processes [8].

Visual Association Areas

Visual association areas, including the occipital and parietal lobes, are responsible for the processing and interpretation of visual stimuli, including written language. These regions communicate with Wernicke's area through intricate neural pathways, enabling the comprehension of written text and the association of visual symbols with meaning [5].

Constituent brain regions

Wernicke's area comprises several distinct anatomical regions, each contributing to its functionality:

Superior Temporal Gyrus (STG)

The superior temporal gyrus is a major component of Wernicke's area. It is involved in the processing of auditory information and is crucial for the comprehension of spoken language. The STG receives input from the auditory cortex and integrates this information with language-related functions.

Supramarginal Gyrus

Situated in the posterior part of the inferior parietal lobule, the supramarginal gyrus is implicated in various aspects of language processing, including phonological processing, articulation, and the interpretation of complex auditory and visual stimuli. It forms an integral part of Wernicke's area, contributing to the comprehension and production of speech.

Angular Gyrus

The angular gyrus, located in the posterior part of the parietal lobe, is involved in language-related functions such as reading, writing, and semantic processing. It is involved in the integration of visual, auditory, and somatosensory information, playing a critical role in reading comprehension and semantic processing. The connection between Wernicke's area and the angular gyrus facilitates the understanding of written language and the retrieval of semantic information from memory [9].

Neural pathways and circuits involved in language comprehension

Language comprehension engages a distributed network of brain regions, including Wernicke's area, Broca's area, the angular gyrus, and other cortical and subcortical structures. The comprehension of spoken and written language involves interconnected processes, beginning with the reception of auditory or visual input, followed by the extraction of linguistic features, and culminating in the assignment of meaning. Auditory language processing begins with the reception of sound waves by the auditory cortex in the temporal lobe. The auditory information is then relayed to Wernicke's area via the primary and secondary auditory pathways, which include the auditory radiations and the superior longitudinal fasciculus (SLF). The SLF serves as a major white matter tract connecting Wernicke's area with other language-related regions, facilitating the integration of auditory information with linguistic knowledge [4]. Visual language processing involves the recognition of written symbols and their conversion into meaningful linguistic representations. Visual stimuli are processed by the occipital lobe and subsequently transmitted to Wernicke's area through the ventral visual pathway, which includes the inferior longitudinal fasciculus (ILF) and the inferior frontal-occipital fasciculus (IFOF) [10].

Wernicke's Area and Language Comprehension

Wernicke's area plays a pivotal role in the comprehension of language, particularly in the processing of auditory and visual linguistic stimuli. Originally described by Carl Wernicke in the late 19th century, this region is associated with the interpretation of language semantics and the integration of auditory and visual information to derive meaning [9]. Semantic processing, involving the understanding of word meanings and their contextual associations, is a fundamental aspect of language comprehension mediated by Wernicke's area. Neuroimaging studies using techniques such as functional magnetic resonance imaging (fMRI) have demonstrated increased activity in Wernicke's area during tasks requiring semantic processing, indicating its involvement in the retrieval and integration of semantic knowledge [11]. Syntax, another essential component of language, refers to the rules governing the arrangement of words and phrases to form grammatically correct sentences. While Broca's area is traditionally associated with syntactic processing and speech production, Wernicke's area contributes to syntactic comprehension by facilitating the parsing and analysis of grammatical structures. Functional connectivity studies have revealed coordinated activity between Wernicke's area and Broca's area during syntactic processing tasks, highlighting their complementary roles in language comprehension [12].

Integration of Semantic and Syntactic Information

The processing of language involves the dynamic integration of semantic and syntactic information to construct coherent linguistic representations. Wernicke's area serves as a convergence zone where semantic and syntactic cues are integrated to facilitate comprehension and interpretation. Studies have shown that damage to Wernicke's area can lead to deficits in both semantic and syntactic processing, resulting in impairments such as fluent aphasia characterized by fluent but meaningless speech [13]. The neurophysiological basis of language comprehension is supported by the complex interactions between Wernicke's area and interconnected brain regions involved in semantic and syntactic processing. Functional neuroimaging studies have provided valuable insights into the neural mechanisms underlying language comprehension, elucidating the contributions of Wernicke's area to the integration of linguistic information across different modalities [3]. In summary, the neurophysiology of language processing involves a distributed network of neural pathways and circuits, with Wernicke's area playing a central role in the comprehension of auditory and visual linguistic stimuli. Through its involvement in semantic and syntactic processing, Wernicke's area contributes to the construction of meaningful linguistic representations, highlighting its significance in the neurobiology of language.

Development of Wernicke's area in infancy and childhood, and its relation to neuroplasticity in recovery from language impairments

Wernicke's area, a critical region of the brain involved in language comprehension, undergoes significant developmental changes during infancy and childhood. Understanding the developmental trajectory of Wernicke's area provides insights into language acquisition and recovery from language impairments, such as Wernicke's aphasia. Moreover, the concept of neuroplasticity elucidates the brain's remarkable ability to reorganize and adapt following injury or impairment. During infancy, the brain undergoes rapid growth and development, including the establishment of key language regions such as Wernicke's area [14]. Although Wernicke's area is not fully functional at birth, its development is influenced by early language exposure and environmental stimuli [15]. As infants are exposed to language through auditory and visual inputs, neural connections within Wernicke's area strengthen, facilitating language comprehension [16]. Through repeated exposure to language, infants develop a rudimentary understanding of phonetic and semantic structures, laying the foundation for later language acquisition [17]. As children progress through childhood, Wernicke's area continues to undergo refinement and specialization [18]. This developmental process is characterized by synaptic pruning, wherein unnecessary neural connections are eliminated while essential connections are strengthened [19]. The maturation of Wernicke's area is influenced by genetic factors, environmental experiences, and language exposure [20]. Additionally, interactions between Wernicke's area and other language regions, such as Broca's area and the angular gyrus, contribute to the integration of language functions [9]. Neuroplasticity, the brain's ability to reorganize and adapt in response to experience, injury, or disease, plays a crucial role in recovery from language impairments [21]. In the context of Wernicke's aphasia, which results from damage to Wernicke's area, neuroplasticity enables the brain to reorganize language functions and compensate for deficits [22]. Following an injury to Wernicke's area, adjacent brain regions may assume language-processing functions through mechanisms such as neural recruitment and functional reorganization [3]. Neuroplasticity in the context of language recovery involves both structural and functional changes within the brain [23]. Structural plasticity encompasses alterations in neural architecture, including dendritic branching, synaptic density, and gray matter volume [24]. Functional plasticity, on the other hand, refers to changes in neural activation patterns and connectivity during language tasks [25]. Through processes such as synaptic plasticity and axonal sprouting, the brain adapts to facilitate the recovery of language abilities following injury or impairment [26]. Recovery from Wernicke's aphasia involves intensive language rehabilitation programs aimed at harnessing neuroplasticity to restore language function [27]. These programs typically incorporate a combination of behavioral therapies, such as speech and language therapy, and technological interventions, including computer-assisted language training [27]. By engaging in repetitive language exercises and tasks, individuals with Wernicke's aphasia can promote neural reorganization and facilitate the recovery of language comprehension and production abilities [28]. In addition to structured rehabilitation programs, environmental enrichment and social support contribute to the promotion of neuroplasticity and language recovery [29]. Engaging in meaningful social interactions and participating in stimulating cognitive activities stimulate neural plasticity mechanisms, enhancing the efficacy of language rehabilitation efforts [30]. Furthermore, multimodal approaches that integrate auditory, visual, and tactile stimuli can maximize neuroplasticity and optimize language outcomes in individuals with language impairments [31]. In conclusion, the development of Wernicke's area during infancy and childhood lays the foundation for language acquisition and comprehension. Understanding the principles of neuroplasticity elucidates the brain's capacity to recover from language impairments such as Wernicke's aphasia. By harnessing neuroplasticity through targeted rehabilitation strategies and environmental enrichment, individuals with language impairments can achieve significant improvements in language function and quality of life.

Current research and future directions in Wernicke's area and language processing

Recent advancements in neuroscience have shed light on the intricate mechanisms underlying language functions and the role of Wernicke's area in these processes. This discussion will summarize recent findings and ongoing research related to Wernicke's area and language processing, while also highlighting emerging technologies and methodologies that may further advance our understanding of this crucial brain region. Recent studies have provided valuable insights into the neural networks associated with Wernicke's area and its involvement in language comprehension. Functional neuroimaging techniques such as functional magnetic resonance imaging (fMRI) have enabled researchers to observe the activation patterns of Wernicke's area during various language tasks. For instance, fMRI studies have revealed increased activation of Wernicke's area during auditory language processing tasks, indicating its role in decoding and understanding spoken language [32]. Moreover, advances in neuroanatomical tracing techniques have elucidated the connectivity patterns of Wernicke's area with other brain regions involved in language processing. Diffusion tensor imaging (DTI) studies have demonstrated the white matter tracts connecting Wernicke's area with Broca's area, a region crucial for language production, highlighting the intricate network underlying language functions [33]. Furthermore, recent research has explored the functional specialization within Wernicke's area and its role in processing different aspects of language. Studies utilizing event-related potentials (ERPs) have provided evidence for distinct neural responses within Wernicke's area to phonological, semantic, and syntactic aspects of language, suggesting functional subdivisions within this region [34]. In addition to investigating the neural basis of language processing, current research endeavors aim to unravel the underlying mechanisms of language disorders associated with dysfunction in Wernicke's area. Conditions such as Wernicke's aphasia, characterized by fluent but nonsensical speech, have prompted researchers to explore the neurobiological underpinnings of language deficits and potential therapeutic interventions [35]. Looking ahead, emerging technologies hold promise for advancing our understanding of Wernicke's area and language processing. One such technology is the application of non-invasive brain stimulation techniques, such as transcranial magnetic stimulation (TMS) and transcranial direct current stimulation (tDCS), to modulate the activity of specific brain regions involved in language functions [36]. These techniques offer potential avenues for therapeutic interventions in language disorders and provide insights into the causal relationships between brain activity and language performance. Furthermore, the integration of neuroimaging modalities, including multimodal imaging approaches combining fMRI, DTI, and electroencephalography (EEG), allows for a comprehensive investigation of the functional and structural aspects of Wernicke's area and its role in language processing [37]. Advanced computational modeling techniques also hold promise for simulating the dynamics of neural networks involved in language functions, facilitating the development of theoretical frameworks to guide empirical research [38].

## Conclusions

In conclusion, recent research efforts have deepened our understanding of Wernicke's area and its involvement in language processing, shedding light on the neural mechanisms underlying language comprehension and production. Ongoing investigations continue to uncover the complexities of Wernicke's area and its functional specialization while emerging technologies offer novel approaches to explore its role in language functions. By integrating multidisciplinary approaches and leveraging cutting-edge technologies, future research endeavors hold the potential to unravel the mysteries of Wernicke's area and pave the way for innovative interventions in language-related disorders.
